# Characterizing Treatment Adherence Trajectories in the endTB Multisite Cohort of Drug-Resistant Tuberculosis Patients: An Application of Group-Based Trajectory Modeling

**DOI:** 10.1093/cid/ciaf467

**Published:** 2025-08-22

**Authors:** Stephanie Law, Isabel Fulcher, Samreen Ashraf, Mathieu Bastard, Wisny Docteur, Molly F Franke, Dalia Guerra, Catherine Hewison, Helena Huerga, Munira Khan, Palwasha Khan, Uzma Khan, Jarmila Kliescikova, Andargachew Kumsa, Nino Lomtadze, Fauziah Asnely Putri, Michael L Rich, Kwonjun Seung, Alena Skrahina, Meseret Tamirat, Luan Nguyen Quang Vo, Carole D Mitnick

**Affiliations:** McGill International TB Centre, Research Institute of the McGill University Health Centre, Montreal, Quebec, Canada; Delfina, San Francisco, California, USA; TB Control Programme–Sindh, National TB Control Programme, Karachi, Pakistan; Global Programme on TB & Lung Health, World Health Organization, Geneva, Switzerland; Partners In Health, Cange, Haiti; Department of Global Health and Social Medicine, Harvard Medical School, Boston, Massachusetts, USA; Partners In Health, Boston, Massachusetts, USA; Socios En Salud Sucursal Peru, Lima, Peru; Médecins Sans Frontières, Paris, France; Epicentre, Paris, France; Interactive Research and Development, Durban, South Africa; Interactive Research and Development Global, Singapore, Singapore; Faculty of Infectious and Tropical Diseases, London School of Hygiene & Tropical Medicine, London, United Kingdom; Interactive Research and Development Global, Singapore, Singapore; Department of Epidemiology, Biostatistics and Occupational Health, McGill University, Montreal, Quebec, Canada; Médecins sans Frontières, Dushanbe, Tajikistan; National TB, Leprosy and Other Lung Diseases Control Program, Addis Ababa, Ethiopia; National Center for Tuberculosis and Lung Diseases, Tbilisi, Georgia; Department of Medicine, David Tvildiani Medical University, Tbilisi, Georgia; Department of Medicine, The University of Georgia, Tbilisi, Georgia; Interactive Research & Development, Jakarta, Indonesia; Partners In Health, Boston, Massachusetts, USA; Division of Global Health Equity, Brigham and Women's Hospital, Boston, Massachusetts, USA; Division of Global Health Equity, Brigham and Women's Hospital, Boston, Massachusetts, USA; WHO Regional Office for Europe, Copenhagen, Denmark; Partners In Health, Boston, Massachusetts, USA; Partners In Health Lesotho, Maseru, Lesotho; Friends for International TB Relief, Ha Noi, Viet Nam; Department of Global Health and Social Medicine, Harvard Medical School, Boston, Massachusetts, USA; Partners In Health, Boston, Massachusetts, USA

**Keywords:** tuberculosis, adherence, group-based trajectory models, directly observed therapy, MDR-TB

## Abstract

**Background:**

In tuberculosis (TB) care, adherence is often assessed using a simple 80% threshold, which may overlook meaningful patterns. We analyzed adherence trajectories among individuals treated for rifampicin- or multidrug-resistant TB (RR/MDR-TB) in the endTB observational study to identify more informative patterns.

**Methods:**

We applied a joint latent class mixed model to classify adherence trajectories and assess their relationship with treatment outcomes. Model performance was compared to common classification methods (eg 80% adherence threshold) using Kendall's τ_b_ and area under the receiver operating curve for predicting unsuccessful outcomes.

**Results:**

Among 1787 individuals, we identified 4 adherence patterns: “consistently high” (72.5%), “high to low” (14.3%), “low to high” (7.3%), and “consistently low” (5.9%). Compared to the “consistently high” group, those in “high to low” (hazard ratio [HR] = 23.2; 95% confidence interval [CI]: 15.7–24.3) and “consistently low” (HR = 43.2; 95% CI: 26.2–71.5) groups had significantly higher risk of unsuccessful outcomes, while the “low to high” group did not (HR = 0.7; 95% CI: .1–3.8). Our trajectory model more accurately predicted outcomes than common classification methods (*P* < .01).

**Conclusions:**

Group-based trajectory modeling provides more nuanced insights into adherence patterns than conventional classification methods. Our findings demonstrate that patients with RR/MDR-TB who exhibited initial poor adherence followed by subsequent improvement achieved clinical outcomes comparable to those with consistently high adherence throughout treatment. This finding challenges the prevailing assumption that sustained high adherence is necessary for treatment success, suggesting that adherence patterns, rather than overall adherence rates, may be more predictive of clinical outcomes in the management of RR/MDR-TB.

## Teaser

Using group-based modeling, we found that people with initially poor but improving adherence to multidrug-resistant TB treatment had outcomes comparable to those with consistently high adherence—challenging the belief that high adherence is required throughout treatment.

## Graphical Abstract

**Graphical Abstract:**
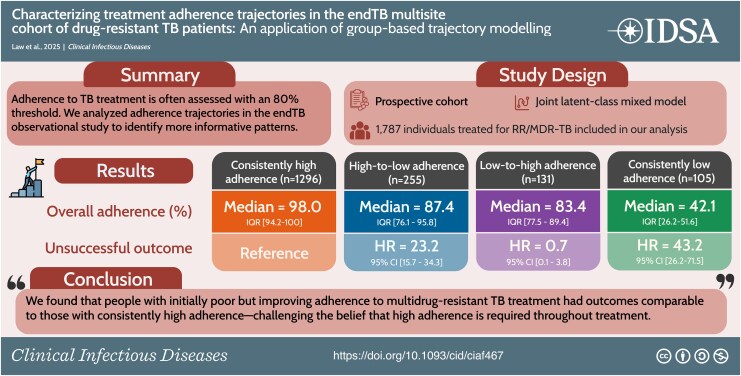
https://tidbitapp.io/institutional-portal/clinical-infectious-diseases/tidbits/characterizing-treatment-adherence-trajectories-in-the-endtb-multisite-cohort-of-drug-resistant-tuberculosis-patients-an-application-of-group-based-trajectory-modelling/update

Globally, tuberculosis (TB) caused illness in 10.8 million people and 1.25 million deaths in 2023 [1]. The emergence of rifampicin-resistant TB (RR-TB) and multidrug-resistant TB (MDR-TB)—resistant to both rifampicin and isoniazid—poses a major threat to ending the global TB epidemic. MDR/RR-TB is found in 3.7% of people newly diagnosed with TB and 18% of those who have been previously treated [1]. Treatment success rates remain below 70%, jeopardizing global efforts to end TB. Adherence is crucial for treatment success, underscoring the global adoption of directly observed therapy (DOT), where patients take each dose under supervision [2, 3]. Yet despite its importance, there is limited research on adherence patterns and their link to outcomes [4].

As MDR/RR-TB regimens shorten, from up to 2 years to as little as 6 months [5], improving adherence may become even more critical, as each dose carries greater weight [6]. In TB care, suboptimal adherence is typically defined using an 80% threshold [6–9]. However, this aggregate measure ignores changes over time, which may affect outcomes differently [10]. Two patients with 80% overall adherence may follow opposite trajectories—one declining over time, the other improving—with potentially different results. Studies have explored the effect of variability in adherence patterns on TB outcomes [11, 12]. Bastard et al [12] found that longer interruptions and shorter gaps between interruptions were linked to poor MDR-TB outcomes; Stagg et al [11] showed that early suboptimal adherence predicted treatment discontinuation in drug-susceptible TB. These findings support the value of more detailed adherence analyses.

We used a group-based trajectory modeling approach [13] to describe adherence patterns and their association with treatment outcomes in the endTB observational study.

## METHODS

### Study Design and Patient Population

**Figure 1.**
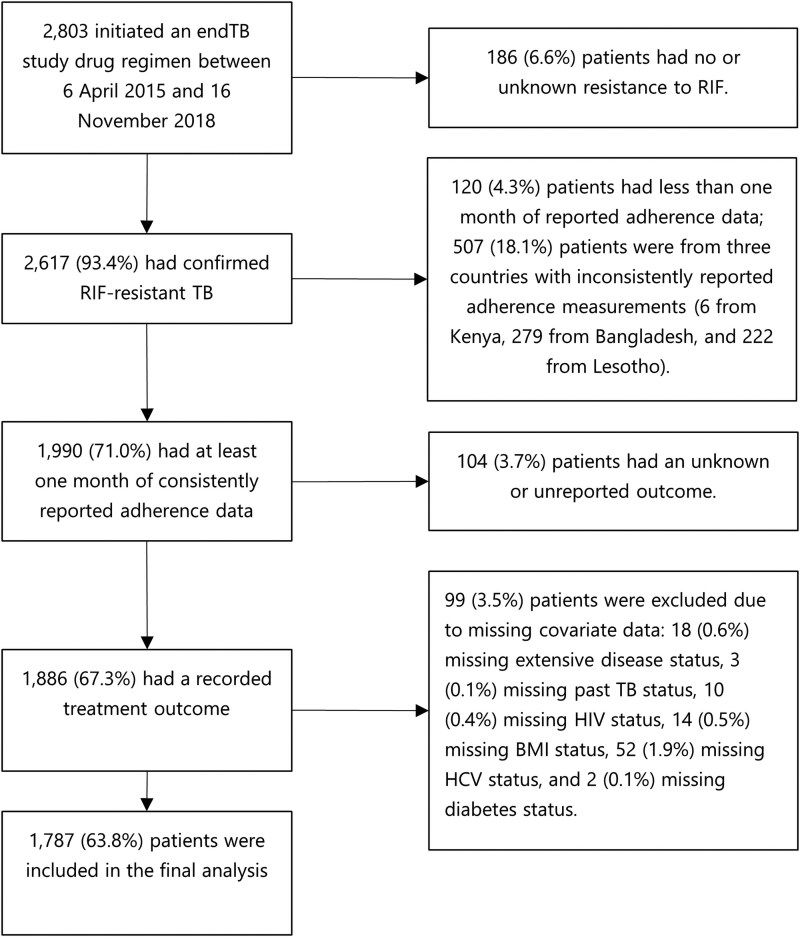
Study flowchart of endTB patients excluded and included in final analysis.

The endTB Observational Study (NCT02754765) is a prospective, multi-country cohort that enrolled patients with MDR/RR-TB whose treatment regimens included at least bedaquiline and/or delamanid, from April 2015 to December 2019 [14]. Treatment regimens were individualized according to relevant national TB guidelines and the endTB clinical guide [15]. Patient recruitment, follow-up and data collection methods have been detailed elsewhere [14]. For this analysis, we included consenting MDR/RR-TB patients who were enrolled in the study, started an endTB regimen after enrollment, had at least 1 month of adherence data, had a recorded final treatment outcome by 1 March 2020, and had complete data on study covariates (Figure 1). We excluded patients from 3 study sites (Kenya, Lesotho, and Bangladesh) due to inconsistent adherence data collection, as reported by site coordinators and study team members.

### Adherence Measurement

Monthly adherence rate was calculated as the number of days in which all medications were taken as prescribed divided by the number of days for which medications were prescribed. The method of recording adherence depended on the treatment delivery method, which included DOT during inpatient or outpatient care, where a health worker observed and recorded whether medications were taken as prescribed, or self-administered therapy, in which adherence was self-reported or recorded via routine pill counts performed by a health worker [14, 16]. Our adherence measure excludes any prescriber-initiated stoppages and interruptions.

### Outcomes and Definitions

Our outcome of interest was any unsuccessful treatment outcome, that is, treatment failure, death, or lost to follow-up, as defined by the WHO [14, 17].

### Statistical Analysis

We calculated descriptive statistics for monthly treatment adherence, baseline covariates, and unsuccessful treatment outcome (failed, died, or lost to follow-up).

**Figure 2.**
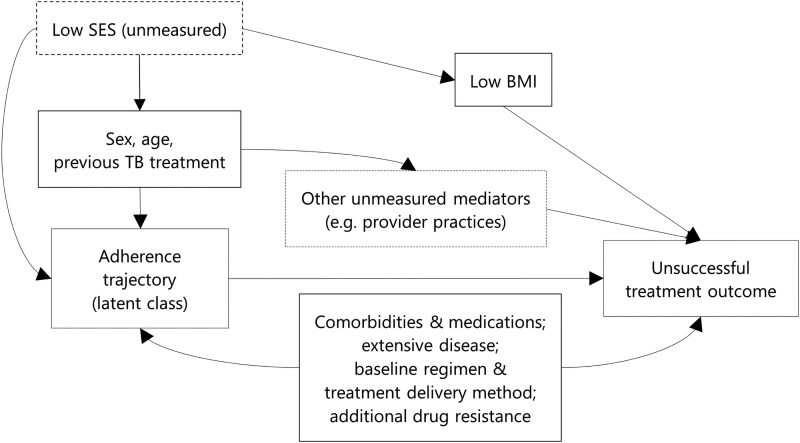
Directed acyclic graph showing relationships between model confounders, unmeasured confounders and mediators, the exposure (adherence trajectory latent class), and outcome (unsuccessful treatment outcome), in the adjusted class-specific survival model within the joint latent class mixed model. Abbreviations: BMI, body mass index; TB, tuberculosis.

We used a joint latent class mixed model to identify adherence trajectories and estimate their association with treatment outcomes. This method assumes homogeneous latent subgroups exist within the heterogeneous population, sharing the same adherence trajectories and event risk [18–20]. Our joint model comprised: (1) a multinomial logistic model estimating probability of latent class membership; (2) a class-specific linear mixed model estimating adherence trajectories; and (3) a class-specific survival model estimating time to unsuccessful outcome. Individuals were classified into the latent class for which their estimated posterior probability is highest. We included baseline characteristics as potential confounders in the survival model: age, sex, previous TB treatment, human immunodeficiency virus/antiretroviral therapy status, hepatitis C virus (HCV) infection, diabetes, extensive disease, low body mass index (<18.5 kg/m^2^), fluoroquinolone resistance, and baseline regimen drugs. A dummy variable accounted for site differences. Confounder inclusion was based on causal relationships to adherence and outcomes (Figure 2). We did not include covariates in the other model components as our objective was to classify adherence trajectories while jointly accounting for time-to-outcome, rather than prediction of adherence.

To select the final model, we compared models with 1–5 latent classes and different link functions (linear, beta, or 1–3 equidistant splines). We assumed proportional hazard Weibull baseline risk. Model adequacy criteria included convergence, minimum 5% class membership and mean posterior probabilities >70% [13, 19]. Among adequate models, we selected that with the lowest BIC.

We compared baseline characteristics and monthly adherence by latent class using χ^2^ and Kruskal-Wallis tests. We reported treatment outcomes by latent class and overall adherence categories (<80% vs ≥80%, quintiles), with adjusted hazard ratios from the joint model.

To compare our approach to standard classification approaches, we estimated Kendall's *τ*_b_ to assess correlation between our latent class subgroups (ordered according to the median overall adherence rates) and: (1) dichotomous and (2) quintiles-based categorizations of overall treatment adherence. To assess whether our approach improves on classification based on overall treatment adherence, we estimated the area under the receiver operator curves (AUROC) for predicting an unsuccessful treatment outcome using each approach, as well as the overall treatment adherence rate as a continuous predictor, and compared them using Delong's method [21].

All statistical analyses were performed using R (version 4.0.0). We estimated the joint latent class mixed model using the *lcmm* package [19].

### Research Ethics

The endTB Observational Study protocol was approved by central ethics review committees for each consortium partner, and local ethical approval was obtained in all endTB countries. Participants provided written informed consent for inclusion in the observational cohort.

## RESULTS

### Overview

Of 2803 consenting individuals in the endTB observational study, we included 1787 (63.8%) in our analysis (Figure 1). Participants received MDR/RR-TB treatment across study sites in Armenia (n = 88; 4.9%), Belarus (n = 89; 5.0%), Ethiopia (n = 37; 2.1%), Georgia (n = 238; 13.3%), Indonesia (n = 68; 3.8%), Kazakhstan (n = 610; 34.1%), Kyrgyzstan (n = 11; 0.6%), Myanmar (n = 39; 2.2%), Pakistan (n = 286; 16.0%), Peru (n = 250; 14.0%), Vietnam (n = 28; 1.6%), and South Africa (n = 43; 2.4%).

The median age was 35 (IQR = 26 to 45) years old, 36.9% were female (n = 660), 65.0% had documented resistance to fluoroquinolones (n = 1093), and 65.7% had extensive disease (n = 1175). Most patients started treatment as inpatients (n = 1089, 60.9%). Other baseline characteristics are shown in Table 1. Median treatment duration was 20 months (IQR 13.5 to 22 months). Median monthly adherence was 95.9% (IQR = 88.8% to 100%); 1539 (86.1%) patients had an overall adherence rate of at least 80%. Overall, 339 (19.0%) patients experienced an unsuccessful treatment outcome: 135 (7.6%) patients died, 59 (3.3%) failed treatment, and 145 (8.1%) were lost to follow-up (LTFU) (Table 2).

**Table 1.** Baseline Characteristics, Treatment Details, and Adherence Rates by Latent Class

	Overall (N = 1787)	Latent Class				
		Consistently High (n = 1296)	High to Low (n = 255)	Low to High (n = 131)	Consistently Low (n = 105)	*P* Value
Baseline characteristics						
Age, median (IQR)	35 (26–45)	34 (25–45)	34 (27–46)	36 (28–48)	39 (30–49)	<.01
Female, n (%)	660 (36.9)	515 (39.7)	83 (32.5)	45 (34.4)	17 (16.2)	<.01
Resistance to fluoroquinolones, n (%)	1093 (65.0)	785 (64.6)	150 (62.8)	86 (67.2)	72 (72.7)	.32
Previous TB treatment, n (%)	1583 (88.6)	1146 (88.4)	227 (89.0)	112 (85.5)	98 (93.3)	.30
Extensive disease, n (%)						
Yes	1175 (65.7)	860 (66.4)	170 (66.7)	82 (62.6)	62 (59.0)	
No	423 (23.7)	314 (24.2)	56 (22.0)	34 (26.0)	19 (18.1)	
Unlikely^a^	190 (10.6)	122 (9.4)	29 (11.4)	15 (11.5)	24 (22.9)	<.01
HIV and ART status, n (%)						
No HIV	1676 (93.8)	1222 (94.3)	237 (92.9)	125 (95.4)	92 (87.6)	
HIV on ART	95 (5.3)	64 (4.9)	15 (5.9)	5 (3.8)	11 (10.5)	
HIV without ART	16 (0.9)	10 (0.8)	3 (1.2)	1 (0.8)	2 (1.9)	.20
Low BMI, n (%)	650 (36.4)	466 (35.0)	107 (42.0)	43 (32.8)	34 (32.4)	.17
HCV, n (%)	177 (9.9)	95 (7.3)	33 (12.9)	20 (15.3)	29 (27.6)	<.01
Diabetes, n (%)	253 (14.2)	186 (14.4)	39 (15.3)	16 (12.2)	12 (11.4)	.71
Treatment details and adherence rates						
Study drugs at baseline, n (%)						
BDQ (without DLM)	1153 (64.5)	836 (64.5)	164 (64.3)	84 (64.1)	69 (65.7)	.09
DLM (without BDQ)	398 (22.3)	275 (21.2)	60 (23.5)	32 (24.4)	31 (29.5)	
Both BDQ and DLM	236 (13.2)	185 (14.3)	31 (12.2)	15 (11.5)	5 (4.8)	
Other drugs at baseline, n (%)						
Moxifloxacin or levofloxacin	1062 (59.4)	777 (60.0)	15.3 (60.0)	81 (61.8)	51 (48.6)	.13
Second-line injectables^b^	851 (47.6)	609 (47.0)	122 (47.8)	73 (55.7)	47 (44.8)	.26
Linezolid	1506 (84.3)	1105 (85.3)	204 (80.0)	112 (85.5)	85 (81.0)	.13
Clofazimine	1351 (75.6)	975 (75.3)	194 (76.1)	98 (74.8)	84 (80.0)	.74
Imipenem^c^ or meropenem^d^	411 (23.0)	260 (20.1)	63 (24.7)	42 (32.1)	46 (43.8)	<.01
Prothionamide or ethionamide	620 (34.7)	466 (36.0)	100 (39.2)	27 (20.6)	27 (25.7)	<.01
Cycloserine	1121 (62.8)	810 (62.5)	149 (58.4)	86 (65.6)	76 (72.4)	.08
Para-aminosalicylic acid	462 (25.9)	306 (23.6)	60 (23.5)	45 (34.4)	51 (48.6)	<.01
Treatment delivery at baseline, n (%)						
Inpatient	1089 (60.9)	800 (61.7)	146 (57.3)	83 (63.4)	60 (57.1)	
Outpatient, community-based DOT	276 (15.4)	233 (18.0)	38 (14.9)	4 (3.1)	1 (1.0)	
Outpatient, facility-based DOT	85 (4.8)	57 (4.4)	17 (6.7)	5 (3.8)	6 (5.7)	<.01
Combination of DOT and SAT	111 (6.2)	81 (6.2)	24 (9.4)	3 (2.3)	3 (2.9)	
SAT	47 (2.6)	41 (3.2)	5 (2.0)	1 (0.8)	0	
Unknown	179 (10.0)	84 (6.5)	25 (9.8)	35 (26.7)	35 (33.3)	
Treatment duration (months), median (IQR)	20 (13.5–22)	21 (16–22)	11 (4.5–19)	21 (20–22)	9 (5–18)	<.01
Monthly adherence rate, median (IQR)	95.9 (88.8–100)	98.0 (94.2–100)	87.4 (76.1–95.8)	83.4 (77.5–89.4)	42.1 (26.2–51.6)	<.01
Overall adherence rate <80%, n (%)	248 (13.9)	14 (1.1)	83 (4.6)	46 (2.6)	105 (100)	<.01
Overall adherence rate, n (%)^e^	…	…	…	…	…	<.01
>98.0%–100%	715 (40.0)	660 (50.9)	55 (21.6)	0	0	
>93.8%–98.0%	357 (20.0)	333 (25.7)	19 (7.5)	5 (3.8)	0	
>85.5%–93.8%	357 (20.0)	246 (19.0)	66 (25.9)	45 (34.4)	0	
0%–85.5%	358 (20.0)	57 (4.4)	115 (45.1)	81 (61.8)	105 (100)	

Abbreviations: ART, antiretroviral therapy; BDQ, bedaquiline; BMI, body mass index; DLM, delamanid; DOT, directly observed therapy; HCV, hepatitis C virus; HIV, human immunodeficiency virus; IQR, interquartile range; SAT, self-administered therapy.

^a^Classified as unlikely extensive disease if no presence of cavity or smear grade <3 but missing the other measurement.

^b^Second-line injectable drugs included amikacin, capreomycin, and kanamycin.

^c^Used in combination with cilastatin and amoxicillin-clavulanic acid, except 2 patients who did not receive the latter.

^d^Used in combination with amoxicillin-clavulanic acid.

^e^Patients in the fourth (>60th to 80th percentile) and fifth quintiles (>80th percentile) all had an overall adherence rate of 100%, therefore there were only 4 categories created based on the quintiles approach.

**Table 2.** Final Treatment Outcomes by Latent Class and Overall Adherence Rate (N = 1787)

	Successful (n = 1448)	Unsuccessful				Adjusted HR (95% CI)
		Any (n = 339)	Died (n = 135)	Failed (n = 59)	LTFU (n = 145)	
Latent class, n (%)						
Consistently high	1208 (93.2)	88 (6.8)	43 (3.3)	32 (2.5)	13 (1.0)	Reference
High to low	84 (32.9)	171 (67.1)	74 (29.0)	15 (5.9)	82 (32.2)	23.2 (15.7–34.3)
Low to high	129 (98.5)	2 (1.5)	1 (0.8)	1 (0.8)	0	.7 (.1–3.8)
Consistently low	27 (25.7)	78 (74.3)	17 (16.2)	11 (10.5)	50 (47.6)	43.2 (26.2–71.5)
Overall adherence rate <80%, n (%)	119 (48.0)	129 (52.0)	29 (35.1)	13 (5.2)	87 (11.7)	not applicable
Overall adherence rate, n (%)	…	…	…	…	…	not applicable
>98.0%–100%	610 (85.3)	105 (31.0)	58 (8.1)	16 (27.1)	31 (4.3)	
>93.8%–98.0%	329 (92.2)	28 (8.3)	14 (3.9)	10 (16.9)	4 (1.1)	
>85.5%–93.8%	305 (85.4)	52 (15.3)	22 (6.2)	16 (4.5)	14 (3.9)	
0%–85.5%	204 (57.0)	154 (43.0)	41 (11.5)	17 (4.7)	96 (26.8)	

Abbreviations: CI, confidence interval; HR, hazard ratio; LTFU, lost to follow-up.

### Description of Adherence Trajectory Latent Classes

**Figure 3.**
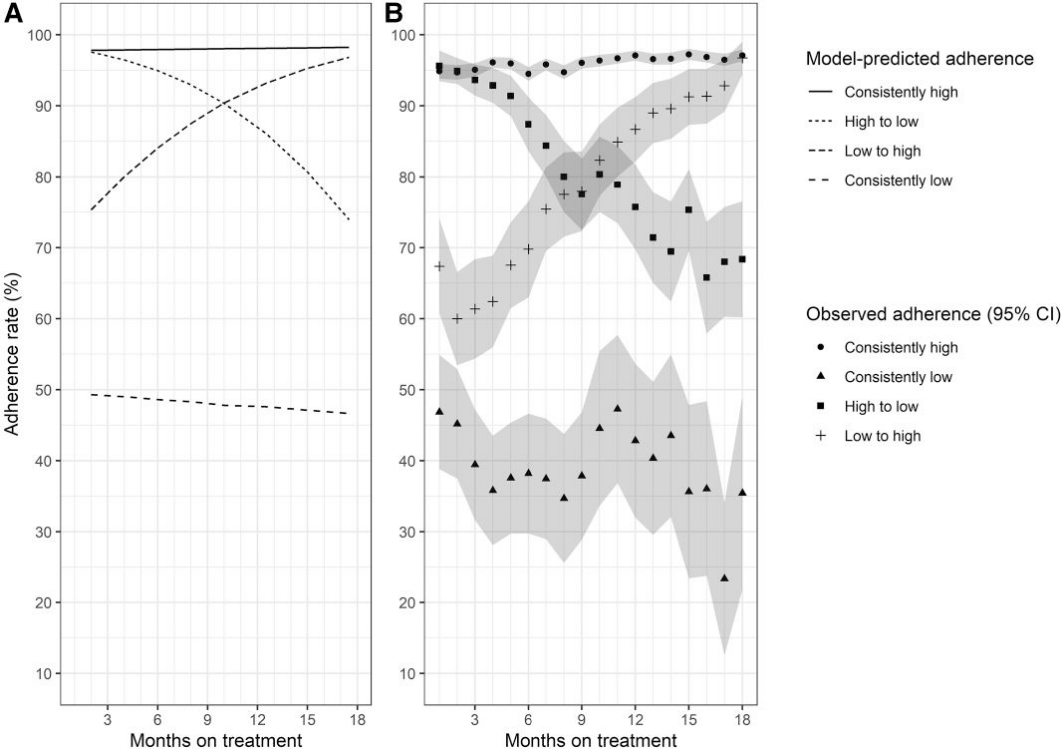
Plots of monthly adherence by latent class: *A*, model-predicted adherence trajectories; *B*, observed average monthly adherence with 95% confidence interval bands. The sample sizes are: consistently high, n = 1296 (72.5%); high to low, n = 255 (14.3%); low to high, n = 131 (7.3%); consistently low, n = 105 (5.9%).

Our final joint latent class mixed model included 1787 individuals with complete data on all model covariates. We identified 4 latent classes of adherence trajectories, which we labeled as: “consistently high” (n = 1296; 72.5%), “high to low” (n = 255; 14.3%), “low to high” (n = 131; 7.3%), and “consistently low” (n = 105; 5.9%) (Figure 3*A*; Table 1). The average posterior probabilities of membership in each class were 85.0%, 95.9%, 85.5%, and 94.6%, respectively. Median monthly treatment adherence was significantly different across the latent classes (*P* < .01), with the highest reported in the “consistently high” subgroup (98.0%, interquartile range (IQR) = 94.2 to 100) and lowest in the “consistently low” subgroup (42.1%, IQR = 26.2%–51.6%) (Table 1); the “consistently low” subgroup also had the lowest initial observed adherence rates (Figure 3*B*). The proportions with an overall adherence rate below 80% were also significantly different across the latent classes (*P* < .01), with the lowest in the “consistently high” subgroup (1.1%) and highest in the “consistently low” subgroup (100%). Among included individuals, 12 were missing adherence data for 1 (n = 11) or 2 (n = 1) months, 8 (61.5%) of whom were from Kazakhstan; everyone else had monthly adherence data for their entire follow-up period. There were no observed patterns of missingness across baseline covariates or latent classes.

### Baseline Patient and Treatment Characteristics by Latent Class

Baseline patient characteristics were similar across the identified latent classes (Table 1), except for age (*P* < .01), sex (*P* < .01), presence of extensive disease (*P* < .01), and having HCV (*P* < .01). The “consistently low” subgroup had the highest median age (39, IQR = 30–49), lowest proportion female (16.2%) and nonextensive disease (18.1%), and greatest proportion with HCV (27.6%). The baseline compositions of treatment regimens were also similar across latent classes (Table 1), except for the use of imipenem or meropenem (*P* < .01), prothionamide or ethionamide (*P* < .01), and para-aminosalicylic acid (PAS) (*P* < .01). The “consistently low” subgroup was more likely to receive imipenem/meropenem and PAS than other subgroups, whereas prothionamide, or ethionamide was more commonly prescribed to the “consistently high” and “high to low” subgroups. Treatment delivery method at the start of treatment was significantly different across the latent classes (*P* < .01), with community-based outpatient DOT found more commonly among the “consistently high” and “high to low” subgroups than the other 2.

### Association Between Latent Classes and Treatment Outcomes

Overall, the proportions of unsuccessful outcomes were lowest in the “low to high” subgroup (1.5%), followed by the “consistently high” subgroup (6.8%), and highest in the “consistently low” group (74.3%) (Table 2). Compared to the “consistently high” subgroup, the relative risk of an unsuccessful treatment outcome was significantly higher in the “high to low” and “consistently low” subgroups, with adjusted hazard ratios of 23.2 (95% confidence interval [CI] 15.7–24.3) and 43.2 (95% CI 26.2–71.5), respectively; there was no significant difference in relative risk in the “low to high” group, with an adjusted hazard ratio of 0.7 (95% CI .1–3.8).

### Comparison of Different Classification Approaches

**Figure 4.**
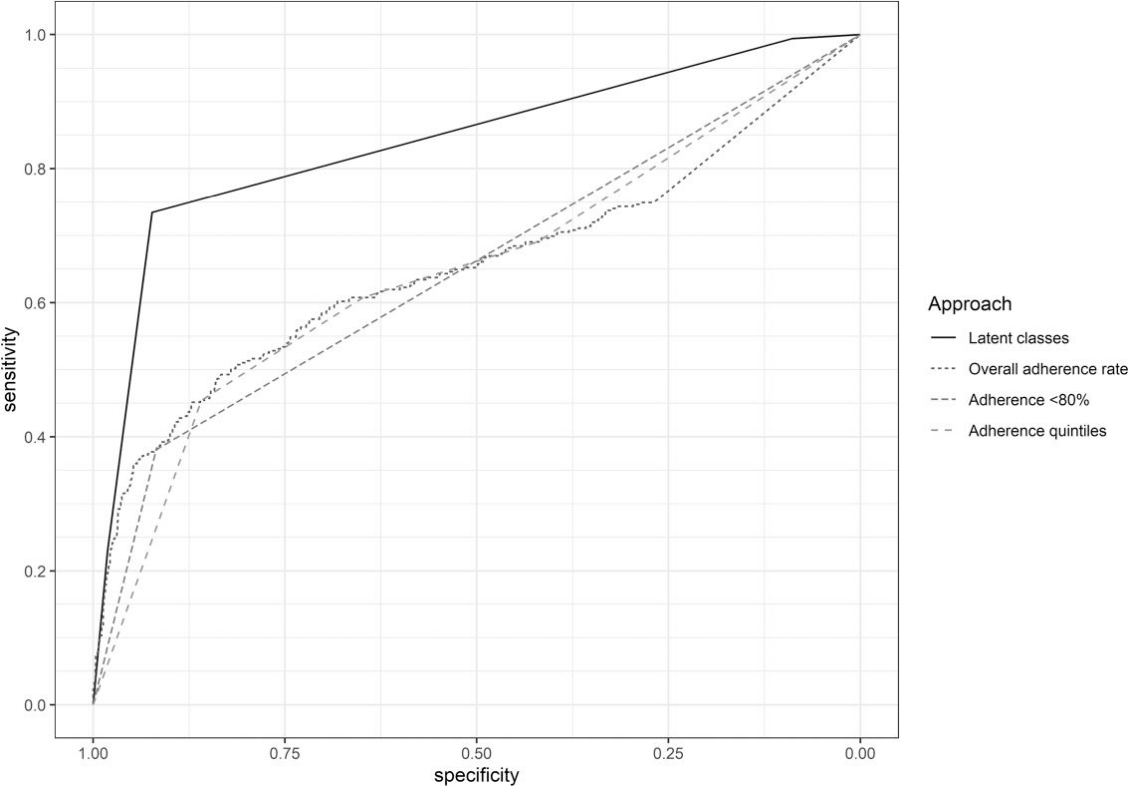
ROC curves for each classification approach. The estimated AUROC for each approach was: latent classes, 0.84 (95% CI .82–.86); overall adherence rate, 0.65 (95%CI .62–.69); adherence rate <80%, 0.65 (95% CI .62–.68); and adherence rate quintiles, 0.65 (95% CI .61–.68). The optimal cutoff for overall adherence rate, estimated using Youden's J Statistic (Youden, 1950), was 87.5%. Using Delong's method, the differences in AUROC between using the latent classes and the other approaches were: 0.19 (95% CI .15–.22), 0.19 (95% CI .16–.22) and 0.19 (95% CI .16–.23), respectively. Abbreviations: AUROC, area under the receiver operating curve; CI, confidence interval; ROC, receiver operating curve.

The estimated Kendall's τ_b_ between the latent classes and dichotomous or quintiles-based categorizations of overall adherence were 0.59 (95% CI .54–.64) and 0.52 (95% CI .49–.55), respectively. Our model-identified latent classes predicted an unsuccessful treatment outcome with higher accuracy than classification approaches based on overall treatment adherence rates (*P* < .01). The estimated AUROCs for the latent class subgroups, overall treatment adherence rate (continuous), adherence rate <80% (dichotomous), and quintiles-based classification, were 0.84 (95% CI .82–.86), 0.65 (95% CI .62–.69), 0.65 (95% CI .62–.68), and 0.65 (95% CI .61–.68), respectively (Figure 4).

## DISCUSSION

Group-based trajectory modeling using latent class mixed models provides a more nuanced characterization of adherence patterns during MDR/RR-TB treatment, while also demonstrating superior predictive capacity for treatment success, compared to conventional classification approaches based on overall adherence rates. Our analysis of a multi-site cohort comprising 1787 patients identified 4 distinct adherence subgroups: “consistently high,” “high to low,” “low to high,” and “consistently low.”

Patients classified within the “consistently high” and “low to high” subgroups demonstrated significantly reduced risk of an unsuccessful treatment outcome relative to the other trajectories. Critically, patients in the “low to high” subgroup—characterized by initial poor adherence followed by improvement—achieved treatment outcomes comparable to those with consistently high adherence, despite exhibiting lower overall adherence than all other subgroups. This finding fundamentally challenges the prevailing assumption that sustained high adherence throughout the treatment course is a prerequisite for optimal outcomes.

These results have important clinical and policy implications. First, they question the current global guidelines’ emphasis on DOT for all individuals initiating treatment, though its cost-effectiveness and equity have been subject to debate [3, 22–24]. Our findings suggest that early adherence may be less critical than previously assumed, and that interventions focused on trust-building, patient education, and counseling may yield superior outcomes [25]. DOT could be more strategically deployed for patients whose adherence deteriorates or fails to improve over time.

Second, early non-adherence should not influence access to socioeconomic support. In some settings, initial poor adherence may limit individuals’ eligibility for community-based care and financial assistance, potentially exacerbating adherence challenges and compromising patient trust [24, 26, 27]. Practices that penalize patients for early non-adherence may paradoxically worsen long-term adherence and treatment outcomes [24, 28–30]. To maximize treatment success rates, patient-centered strategies that avoid penalizing early poor adherence while actively supporting adherence improvement are essential.

Our analysis identified several baseline characteristics associated with adherence trajectory membership. Male patients, older individuals, and those with non-extensive disease or hepatitis C co-infection, were more likely to belong to subgroups associated with poorer treatment outcomes, while patients with extensive baseline disease were more likely to achieve favorable adherence patterns—findings consistent with research in other chronic conditions [31]. Certain baseline medications (imipenem, cilastatin, meropenem, amoxicillin-clavulanate, cycloserine, or PAS) were more prevalent in the “consistently low” subgroup, potentially reflecting tolerability or access barriers. These baseline differences support the development of targeted interventions for specific patient subgroups.

Our study had several limitations. First, adherence data collection varied by site and delivery method—facility-based DOT involved direct observation, while self-administered therapy relied on self-report or pill counts, potentially overestimating adherence. Still, consistently high adherence was linked to better outcomes. Second, we did not account for time-varying confounding, opting for a simpler model to describe adherence trajectories. Despite this, our joint model includes a time-to-event survival sub-model to account for correlation between adherence and outcomes over time, and suggests these trajectories are meaningfully associated with outcomes and may be predicted by baseline characteristics. Future work should explore potential baseline and time-varying predictors of adherence trajectories and test for interactions. Third, we did not explore causal pathways to explain the association between adherence patterns and treatment outcomes; future studies should identify potential explanations for these relationships. Finally, we used complete case analysis; the effect of this on our analysis is likely negligible since the overall amount of missingness in model covariates was low (3.5%).

Our study adds to the limited literature examining adherence patterns—beyond the use of binary thresholds (eg 80% cutpoint or treatment completion)—among people treated for TB [11, 12, 32]. Our findings align with those by Bastard et al [12], in that if adherence remains poor over a long period, unsuccessful MDR/RR-TB outcomes become significantly more likely. Our findings however contrast those by Stagg et al [11], who found poor adherence early-on predicted treatment discontinuation among people with drug-susceptible TB. This suggests that longer treatment duration and intensified clinical monitoring for MDR/RR-TB might provide opportunities to improve adherence and final treatment outcomes. Finally, Huddart et al [32] similarly applied a group-based trajectory model to early adherence (first 12 weeks) in a small, single-site MDR/RR-TB cohort and found limited predictive value beyond a 90% cutpoint. By comparison, our model used data from a large, multisite study covering the full treatment course. It more accurately predicted outcomes than conventional adherence thresholds. This suggests early adherence alone is insufficient for predicting MDR/RR-TB outcomes and that arbitrary cutpoints commonly used in TB research fail to capture important adherence dynamics over time [7, 10].

This research provides compelling rationale for collecting comprehensive, longitudinal adherence data in future TB treatment studies, particularly as treatment regimens are shortened and adherence impact on outcomes intensifies [6]. Future research priorities should include time-varying causal analyses to identify mechanistic pathways and optimal intervention timepoints, qualitative investigations to elucidate adherence pattern determinants, and randomized controlled trials evaluating intervention effects on long-term adherence trajectories.

## CONCLUSIONS

Current approaches to TB treatment adherence analysis predominantly employ dichotomous classifications based on overall adherence thresholds (typically 80%), an oversimplified methodology that fails to capture the complexity of real-world adherence behaviors and provides insufficient guidance for developing targeted intervention strategies. Our study demonstrates that group-based trajectory modeling can overcome these limitations by enhancing treatment outcome prediction and providing comprehensive insights into patient adherence patterns throughout the treatment course.

Most significantly, our findings reveal that patients exhibiting initial poor adherence who subsequently achieved adherence improvement had treatment success rates comparable to those maintaining consistently high adherence throughout therapy. This finding fundamentally challenges the widely accepted paradigm that TB cure requires high adherence rates (such as the commonly cited 80% threshold) maintained consistently across the entire treatment duration.

These results suggest that adherence trajectory patterns, rather than overall adherence rates, may be more clinically relevant for predicting treatment success, with important implications for both clinical practice and adherence support strategies.
